# The prostate after castration and hormone replacement in a rat model: structural and ultrastructural analysis

**DOI:** 10.1590/S1677-5538.IBJU.2016.0484

**Published:** 2017

**Authors:** Bruno Felix-Patrício, Alexandre F. Miranda, Jorge L. Medeiros, Carla B. M. Gallo, Bianca M. Gregório, Diogo B. de Souza, Waldemar S. Costa, Francisco J. B. Sampaio

**Affiliations:** 1 Instituto de Ciências Humanas e da Saúde, Universidade Federal Fluminense, Rio das Ostras, RJ, Brasil; 2 Urogenital Research Unit, Universidade Estadual do Rio de Janeiro, Rio de Janeiro, RJ, Brasil; 3 Fundação Educacional Dom André Arcoverde, Valença, RJ, Brasil

**Keywords:** Hormone Replacement Therapy, Prostate, Orchiectomy, Hypogonadism

## Abstract

**Purpose:**

To evaluate if late hormonal replacement is able to recover the prostatic tissue modified by androgenic deprivation.

**Materials and Methods:**

24 rats were assigned into a Sham group; an androgen deficient group, submitted to bilateral orchiectomy (Orch); and a group submitted to bilateral orchiectomy followed by testosterone replacement therapy (Orch+T). After 60 days from surgery blood was collected for determination of testosterone levels and the ventral prostate was collected for quantitative and qualitative microscopic analysis. The acinar epithelium height, the number of mast cells per field, and the densities of collagen fibers and acinar lumen were analyzed by stereological methods under light microscopy. The muscle fibers and types of collagen fibers were qualitatively assessed by scanning electron microscopy and polarization microscopy.

**Results:**

Hormone depletion (in group Orch) and return to normal levels (in group Orch+T) were effective as verified by serum testosterone analysis. The androgen deprivation promoted several alterations in the prostate: the acinar epithelium height diminished from 16.58±0.47 to 11.48±0.29μm; the number of mast cells per field presented increased from 0.45±0.07 to 2.83±0.25; collagen fibers density increased from 5.83±0.92 to 24.70±1.56%; and acinar lumen density decreased from 36.78±2.14 to 16.47±1.31%. Smooth muscle was also increased in Orch animals, and type I collagen fibers became more predominant in these animals. With the exception of the densities of collagen fibers and acinar lumen, in animals receiving testosterone replacement therapy all parameters became statistically similar to Sham. Collagen fibers density became lower and acinar lumen density became higher in Orch+T animals, when compared to Sham. This is the first study to demonstrate a relation between mast cells and testosterone levels in the prostate. This cells have been implicated in prostatic cancer and benign hyperplasia, although its specific role is not understood.

**Conclusion:**

Testosterone deprivation promotes major changes in the prostate of rats. The hormonal replacement therapy was effective in reversing these alterations.

## INTRODUCTION

Androgen deficiency of the aging male (ADAM) is a well described syndrome which affects 6% of 40 years old men and 12.3% of 69 years old men (1). Hormonal replacement therapy is efficacious in establishing normal testosterone serum levels and relieving clinical symptoms, improving libido, sexual function, mood and reducing fat body mass (2).

Despite the beneficial aspects of testosterone replacement, the hormone replacement therapy is not completely accepted, due to its possible collateral effects and risks (3). Because of the fear of developing prostatic diseases, or because of poor medical assistance, many men do not begin replacement therapy until testosterone level is very low and clinical symptoms established (4).

The prostate is an androgen-dependent organ and as so, is influenced by the testosterone serum levels. Androgen ablation in adulthood promotes a fast and extensive involution of the prostate with arrest of secretory activity and elimination of epithelial cells by apoptosis (5). The prostate extracellular matrix is also modified by low testosterone levels. In an experimental model with chemically or surgically castrated gerbils, increase in collagen fibers and fibromuscular stroma was observed (6). The extracellular matrix is also altered in patients with benign prostatic hyperplasia, with increased densities of reticular fibers and fibronectin (7, 8). Although this information was not correlated with testosterone levels, it was shown that the prostatic stroma of old mice has also several modifications, with abundant and disorganized collagen fibers, and disordered smooth muscle orientation (9).

Mast cells are enrolled in many biologic responses through degranulation and secretion of its contents. It is well known that these cells plays a role in chronic inflammation, hemotopoiesis, hemostasis, angiogenesis, tissue remodeling, and fibrosis (10). Furthermore, mast cells control and induce the extracellular matrix production and deposition in certain fibroproliferative diseases (11). It was shown that the amount of mast cells increases from birth to youth adulthood in the human prostate (12), and these cells were implicated in benign prostatic hyperplasia (10, 13), as well as in prostate cancer, being even proposed as a prognostic marker (14, 15). A great amount of mast cells were also reported in the prostate of rats (16). Its density was highest during the pubertal period, declining significantly with age (17). However, the direct influence of testosterone levels on prostate mast cells was not previously investigated.

Several methods have been used for studying the human and animal prostate in different conditions. Although conventional light microscopy gives important information on the prostate epithelium and stromal characteristics, the quantification of these structures by morphometrical methods may translate the tissue characteristics into numbers, what allows statistical comparison among different groups (18, 19). Further, scanning electron microscopy and polarization microscopy can be used to distinguish collagen types (20) and its three-dimensional patterns (21-23). Scanning electron microscopy has been used both with cellular digestion pre-treatment, whereas the connective tissue is very well depicted (21, 22), and without this treatment for observation of cellular components (23). Sirius red stained sections observed under polarization microscopy shows a birefringent image that is highly specific for collagen, and allows the differentiation of collagen types. This characteristic has been used to investigate the collagen in the prostate (24, 25) as well as in other organs (26, 27).

Nevertheless, the influence of late hormonal replacement over the prostate of individuals with low testosterone levels is not known. Thus, the objective of the present study is to evaluate in a rat model if the late hormonal replacement is able to recover the prostatic tissue modified by androgenic deprivation.

## MATERIAL AND METHODS

### 

#### Animals

Twenty four Sprague-Dawley male rats aged 24 weeks were included in the present study. The rats were kept in a room with controlled temperature (24±1°C), artificial dark-light cycle (lights on from 7:00 am to 7:00 pm) and standard rat food and water ad libitum.

All experiments were performed in accordance with the Brazilian laws for scientific use of animals, and the project was approved by the ethical committee for the care and use of experimental animals, of the Institute of Biology Roberto Alcantara Gomes, State University of Rio de Janeiro (protocol number 231/2008).

#### Groups

The rats were randomly assigned into a Sham group (n=8), which was submitted only to anesthesia and a simulated operation, an androgen deficient group (Orch; n=8), submitted to bilateral orchiectomy, and a group with hormone replacement (Orch+T; n=8) which was submitted to bilateral orchiectomy followed by testosterone replacement therapy.

#### Surgical procedure

Under ketamine (80mg.Kg^−1^) and xylazine (10mg.Kg^−1^) anesthesia, bilateral orchiectomy was performed through a scrotal exposure. Testes were exposed and removed after spermatic cords ligature in groups Orch and Orch+T. Group Sham were submitted only to testes exposure and reinsertion into the scrotum. For all animals, scrotal incision was closed with 4-0 nylon in a simple interrupted pattern.

#### Hormone replacement

Animals from Orch+T group received a single-dose subcutaneous injection of testosterone 30 days after surgery. Replacement therapy was done according to a previously described protocol (28-30), using of testosterone undecanoate (Jenahexal Pharma, Jena, Germany) at 100mg/kg body weight.

#### Euthanasia and tissue collection

After 60 days from surgery, under deep anesthesia, blood was collected by heart puncture and the serum was separated by centrifugation and used to testosterone determination by radioimmunoassay. The rats were killed by anesthetic overdose and the prostate was en bloc removed. The ventral lobe, which better corresponds to human prostate (31, 32), was then dissected under magnification and divided in two fragments for different analysis.

#### Light microscopy analysis

Fragments used for light microscopy analysis were fixed in 4% formaldehyde and routinely processed for obtaining 3μm thickness sections. Images of conventional and polarized light microscopy were captured by a DP71 camera coupled to BX51 microscope (Olympus, Tokyo, Japan). Images of fluorescent microscopy were obtained in 400x magnification by a scanning laser confocal microscope (LSM 510 META, Zeiss, Jena, Germany)

#### Acinar epithelium height

The acinar epithelium height was measured in hematoxylin & eosin stained sections. The linear distance from the luminal surface of the glandular epithelium and its basement membrane was measured in micrometers. For this purpose, the ImageJ software version 1.43 (NIH, Bethesda, Maryland, USA) was used with previously calibration for the magnification of 600X. The mean of each animal was calculated after 250 measurements performed in at least 25 different fields.

#### Density of acinar lumen

The area density of acinar lumen (Sv[lum]) was calculated by the point-counting method in the same sections. A 100-point grid was superimposed over the images using the software ImageJ, and the acinar lumen touched by the points were counted (33). For each animal, Sv[lum] was determined by the mean of measurements in 25 fields with 100X magnification.

#### Mast cells quantification

The number of mast cells was accessed in toluidine blue stained sections, under a 600X magnification. For this analysis, at least 50 fields were analyzed in order to calculate each animal mean. Again, ImageJ software was used for this measurement. For this purpose, in each image (histological field) all mast cells were manually counted with aid of the “cell counter” plugin. This parameter was expressed as mast cells per field.

#### Qualitative analysis of muscle fibers

Muscle fibers were qualitatively analyzed in anti α-actin fluorescent immunolabeled sections under 400X magnification. Antigen retrieval was carried out prior to incubating the sections with the primary antibody by treating dewaxed sections with a ready-made pepsin solution (Digest-All Kit, Zymed Laboratories, San Francisco, California, USA), according to the manufacturer's instructions. The primary antibody used was a monoclonal anti-α-smooth muscle-actin (08-0106, Zymed Laboratories, Carlsbad, California, USA). Secondary antibody was Alexa Fluor 488 (A-1100, Invitrogen, Camarillo, California, USA).

#### Density of collagen fibers

The area density of collagen fibers (Sv[col]) was measured in Sirius red stained sections, observed under polarized light at a magnification of 200X, in which only collagen appears birefringent over a dark field (20). Image pro plus was used for measuring Sv[col] by color segmentation. These same images were used to differentiate collagen types III (seen in green) and I (red/ orange) (20).

#### Electron microscopy analysis

The differences of collagen fibers were also analyzed by scanning electron microscopy (SEM). For this purpose, samples were fixed by immersion in 2.5% glutaraldehyde and cellular content was chemically removed by alkali treatment (34). After this treatment, samples were dehydrated in ethanol, critical point-dried with CO_2_ and sputter-coated with gold for observation in a LEO 435 (Carl Zeiss, Oberkochen, Germany) scanning electron microscope for differences on the thickness and arrangement of collagen fibers (21).

### Statistical analysis

The one-way ANOVA, followed by Bonferroni's multiple comparison test were used for mean comparisons. In all cases, significance was considered when p<0.05. All analyzes were performed using GraphPad Prism software (GraphPad Software, San Diego, California, USA). All numerical data is present as mean±standard deviation.

## RESULTS

### 

#### Testosterone analysis

Hormone depletion and replacement were effective as verified by serum testosterone analysis. Animals from group Orch had undetectable levels of testosterone while Orch+T rats had statistically similar values of this hormone when compared to Sham (Table-1).

**Table 1:** Hormonal and morphometric data of prostate from rats of groups Sham (n=8), hypogonadal (Orch) (n=8), and hypogonadal with hormonal replacement (Orch+T) (n=8).

Groups	Sham	Orch	Orch+T	p value
Testosterone level (ng/mL)	1.28±0.28	0.00±0.00	0.99±0.23	<0.0001
Epithelium height (μm)	16.58±0.47	11.48±0.29 a	17.67±1.26 b	<0.0001
Sv[lum] (%)	36.78±2.14	16.47±1.31 a	43.18±4.46 a,b	<0.0001
Mast cells (cells/field)	0.45±0.07	2.83±0.25 a	0.52±0.14 b	0.0024
Sv[col] (%)	5.83±0.92	24.70±1.56 a	2.81±0.19 a,b	<0.0001

**Sv[lum]**, area density of acinar lumen; **Sv[col]**, area density of collagen fibers. Post-test analysis:

a ≠from Sham group;

b ≠from Orch group.

Data is present as mean±standard deviation.

#### Quantitative morphological analysis

The acinar epithelium height of Orch animals was of 11.48±0.29μm, thus it was diminished (p<0.0001) in comparison to Sham animals which presented values of 16.58±0.47μm. This alteration was not present in Orch+T in which there was no statistical difference from Sham (Table-1).

Regarding Sv[lum], Orch animals presented a significant decrease (p<0.001) in comparison to Sham. Orch animals had values of 16.47±1.31%, while Sham animal's values were of 36.78±2.14%. Hormonal replacement therapy was effective in recovering of Sv[lum]. Actually, animals that received the therapy presented values of 43.18±4.46%, which was even higher than those of Sham animals (Table-1).

**Figure 1:**
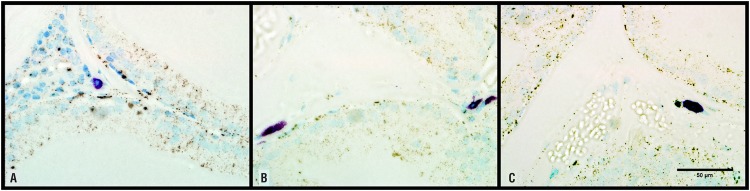
Ventral prostate of rats stained with toluidine blue and observed 600X magnification. In image A) we observe normal quantity of mast cells (0.45±0.07) per field. In image B) (group Orch) we observe an increase on the number of mast cells per field (2.83±0.25), which returned to normal (0.52±0.14) in animals receiving hormonal replacement as seem in image C) (group Orch+T).

The number of mast cells per field in group Orch was 2.83±0.25, that was increased (p=0.0024) in comparison to Sham animals, that presented 0.45±0.07 mast cells per field (Figure-1). Between Sham and Orch+T groups no significant difference was found (Table-1).

Concerning Sv[col], Orch animals presented a significant increased stromal collagen in comparison to Sham animals (p<0.001). Orch animals had values of 24.7±1.56%, while Sham animal's values were of 5.83±0.92%. For this parameter, hormone therapy promoted a decrease of the collagen content, returning to 2.81±0.19% (Table-1).

#### Qualitative morphological analysis

**Figure 2:**
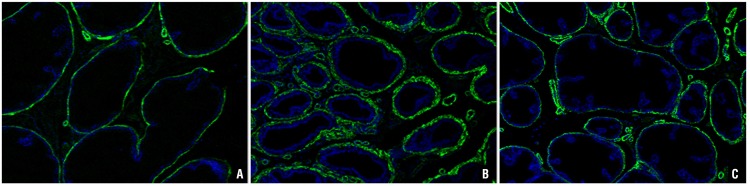
Ventral prostate of rats immunolabeled with anti α-actin antibodies and observed under fluorescence microscopy under 400X magnification. In image A) we observe normal quantity of smooth muscle of Sham group. In image B) (group Orch) we observe an increase of muscle fibers, which returned to normal in image C) (group Orch+T).

The amount of periacinar smooth muscle fibers showed was marked increased in group Orch. This alteration was not noted in Orch+T animals (Figure-2).

When observed under polarized light, the Sirius red stained sections showed an equivalent amount of types I and III collagen in Sham, which has changed to a predominant red pattern in Orch sections, indicating a major presence of type I collagen. Sections of Orch+T presented a very similar pattern of those from Sham group.

**Figure 3:**
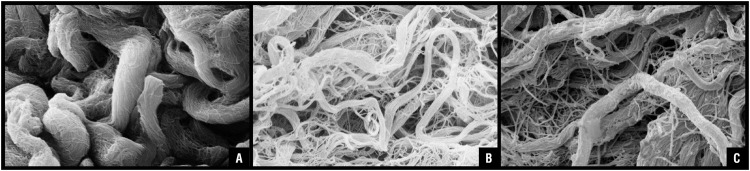
Decellularized ventral prostate of rats observed under scanning transmission microscopy. In image A) (group Sham) we observe both thin and thick collagen fibers, compatible with types I and III, respectively. In image B) from group Orch, there is a predominance of type I collagen fibers, while image C) (group Orch+T) resembles what was observed in image A, with presence of both collagen types.

Similar modifications of collagen fibers were also seen when observing acellular prostate preparations by scanning electron microscopy. With this method, it was observed both thin and thick collagen fibers in group Sham, compatible with types I and III, respectively. In group Orch, it was found a predominance of type I collagen fibers, while in group Orch+T it was observed an equivalent presence of both collagen types (Figure-3).

## DISCUSSION

ADAM is a syndrome that should be not underestimated as it implicates in patient's life quality. Besides its genital and psychological effects, systemic diseases (such as obesity, diabetes and hypertension) are related to hypogonadism (35). Even so, more than 68% of physicians believe that hormonal therapy for ADAM implies in more risks than benefits (4). The concern that testosterone therapy may induce benign prostatic hyperplasia or prostate cancer was proven not to be true (4, 36). Actually, testosterone replacement improves mood and feelings of well-being, sexual function, muscle mass and strength, bone density, and reduction of body fat mass and waist circumference (37). Even so, 35% of patients with diagnosed ADAM do not receive treatment because of the risks of hormonal therapy (4).

Under low levels of testosterone, it is well recognized that the prostate suffers structural changes, with modifications in its acinar epithelium, basement membrane, elastic system fibers and collagen (5, 38-40). However, the effects of hormone replacement therapy are still fairly known. In the present study, late testosterone replacement (30 days after orchiectomy) was able to overturn most of the changes associated with hypogonadism, increasing the height of the acinar epithelium (which was reduced by 30% in castrated animals) and acinar lumen area density (reduced by 55% in castrated animals). Both these parameters are linked to prostate physiology since they indicate normal prostatic fluid production. Corroborating with these data, some clinical studies suggests that after testosterone therapy the prostate volume increases, usually to the normal volume seen in eugonadal individuals (2).

We should point out that the testosterone reposition therapy in men is still a controversial topic, especially in the men with a history of prostate cancer (41). The main concern is that testosterone may accelerate prostate growth not only in benign conditions but also in cancer. Although the current knowledge indicates that testosterone reposition does not appear to increase PSA levels or the risk of prostate cancer development, studies with longer follow-up are still being performed, and the topic is still on discussion (42).

The prostate gland should not be seen only as a site for diseases. Its secretion accounts for 30% of the seminal plasma (43) which actively participates on the sexual and reproductive process. The prostatic fluid not only carries the spermatozoa into the female reproductive tract, but its components participate in key events related to sperm function, fertilization and embryo development in the female reproductive tract (44, 45). A histologically normal prostate is necessary for normal prostatic production and thus may be desirable for normal sexual and reproductive function in ADAM patients.

Besides the glandular epithelial cells, other cell types are present on prostate. Numerous mast cells are observed in the stroma of the rat ventral prostate, and they are often observed close to blood vessels and nerves in the prostate as well as in other organs (17). In the present study, we observed that castration induced a 528% increase in mast cells in the ventral prostate which was overturned by hormonal therapy. Little information is known about the influence of testosterone and mast-cells. It was shown that mast cells present in the Harderian gland of hamsters are “androgen-dependent” cells. These mast cells are induced to degranulate when testosterone links to its specific receptors (46). Other study demonstrated that mast cells from human foreskin and breast skin (from female subjects) express androgen receptor. These authors showed that male skin mast cells expresses (10.8-fold) higher levels of androgen receptor than those from females (47). It seems that testosterone may influence the mast cells of some regions, although this was not proven yet. The recent information correlating the mast cell density with the prognostic of prostate cancer (14) corroborates this hypothesis. Future studies that address the influences of testosterone on mast cells and its role in normal and pathological prostate conditions are warranted.

The testosterone depletion is also linked to increased collagen deposition and augmented smooth muscle fibers in the human (48) and animal prostate (5, 28, 38, 39, 49). An accumulation of collagen fibers and smooth muscle cells around the rat prostate epithelium after castration was previously reported (50), and confirmed in the present study. Collagen density was augmented by 323% in castrated rats. Also, it was shown that the collagen has different characteristics as seen by optical and electron microscopy what is indicative of a turnover of this structure. Most importantly, the hormonal replacement therapy was effective in reversing (partially or completely) these stromal alterations.

We should point out that this is an animal study and its results should not be directly transposed to humans. Although the rodent model has been very well accepted for these studies, this is still an experimental setting and different from the clinical setting. Further, these castrated animals were healthy individuals, without any other medical condition which, again, does not represents the majority of patients with hypogonadism.

## CONCLUSIONS

The present study demonstrates that the prostate of castrated rats suffers major morphological modifications, with parenchymal reduction and increase of stromal structures, including a higher presence of mast cells. The testosterone replacement restores the original architecture of prostate, regarding both parenchyma and stromal structures.
